# Studies of Limit of Detection on 2,4,6-Trinitrotoluene (TNT) by Mass Spectrometry

**DOI:** 10.6028/jres.093.105

**Published:** 1988-06-01

**Authors:** M. R. Lee, S. C. Chang, T. S. Kao, C. P. Tang

**Affiliations:** Chung Shan Institute of Science and Technology, P.O. Box 1-4, Lung-Tan, Taiwan, R.O.C.

Various ionization methods including positive chemical ionization (PCI), negative chemical ionization (NCI) and electron impact (EI) were used to study the mass spectra of TNT. Methane, isobutane and ammonia were used as the CI reagent gases. The mass spectrometric quantitation in this study was performed by selected ion monitoring (SIM), with sample introduction via a short capillary column and a solids probe. The best TNT detection limit (ca. 0.020 ng) was obtained with the NCI-SIM technique with isobutane as a reagent gas.

## Introduction

The trace analysis of explosives is of importance in forensic science and analytical problems encountered in this field involve the detection of nanogram quantities of explosives in extracts obtained from post-explosion residues [1]. The identification of an explosive residue usually involves extracting the debris with acetone or methanol, then separating the extract by chromatographic methods coupled with a detection technique. Single ion monitoring (SIM) by combined gas chromatography-mass spectrometry is the most promising technique to determine a trace amount of an explosive in an unknown mixture.

The mass spectra of a series of explosives have been reported [2–3]. This report describes the investigation of the limit of detection (LOD) of TNT by gas chromatography-mass spectrometry.

## Experimental

All mass spectra were generated with a Finnigan Model 4023 combined gas chromatograph/mass spectrometer (GC/MS) equipped with a dual CI/EI source. Ultra-high purity methane, isobutane and ammonia (Matheson, Morrow, GA) were used as CI reagent gases.

A 1000 ppm solution was prepared by dissolving purified TNT in acetone, then diluted to different desired concentrations as standard sample solutions. TNT was eluted on a two-meter-long fused silica capillary column (Supelcowax 10) using a helium carrier at 8 psig head pressure. The GC temperature was programmed from 80–250 °C at 30 °C/min. Triplicate 1.0 *μ*L injections of each sample were made.

To operate the solid-probe, the temperature was kept at 40 *°*C for 2 minutes then heated to 60 °C directly. Triplicate 1.0 *μ*L samples of a series of standard solutions were injected into separate 5 *μ*L glass vials, allowed to air dry, and then introduced into the ion source via the solid-probe.

The selected ions monitored in different modes and the optimized quantitation conditions are listed in table 1. The quantification signal was obtained by the GC peak area, which was the integrated ion current during elution of TNT.

## Results

For the quantitative studies, the limit of detection (LOD) was calculated as the amount of sample necessary to give a signal-to-noise (S/N) ratio of 3. Based on the results of a series of standard TNT solutions, the calibration curves were constructed. From these calibration curves the limit of detection for EI, PCI and NCI with CH_4_, i-C_4_H_10_ and NH_3_ as reagent gases were calculated and are tabulated in table 1. The relative standard deviations of integrated signals for the triplicate analyses having S/N ratio greater than 3 ranged from 5% to 25% for the PCI technique and 2% to 17% for NCI.

**Figure 1:**
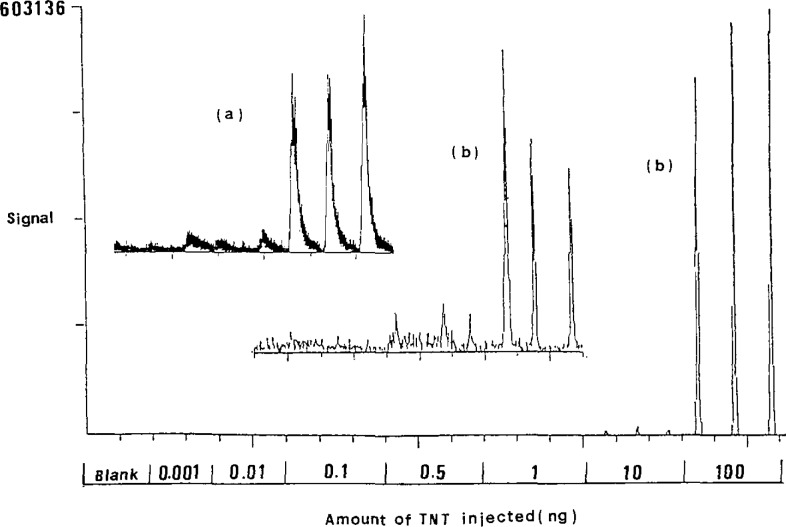
Quantitation of TNT by short capillary column a) NCI(i-C_4_H_10_)–SIM(227), b) NCI(CH_4_)–SIM(227).

From table 1 and figure 1, it was found, as reported [4], that except for NH_3_–NCI, the LOD by CI is at least one order lower than that by EI. Regardless of reagent gas used, the ion currents under electron capture conditions in the negative mode exceeds that in the positive ion mode by one or two orders of magnitude. The most sensitive result, an LOD of 0.020 ng was obtained in i-C_4_H_10_–NCI mode as shown in figure 1. Perhaps the energy transfer with isobutane is much less than with methane, and thus causes less fragmentation of the TNT molecular ion (M^7^) which was selected as the monitored ion.

**Figure 2:**
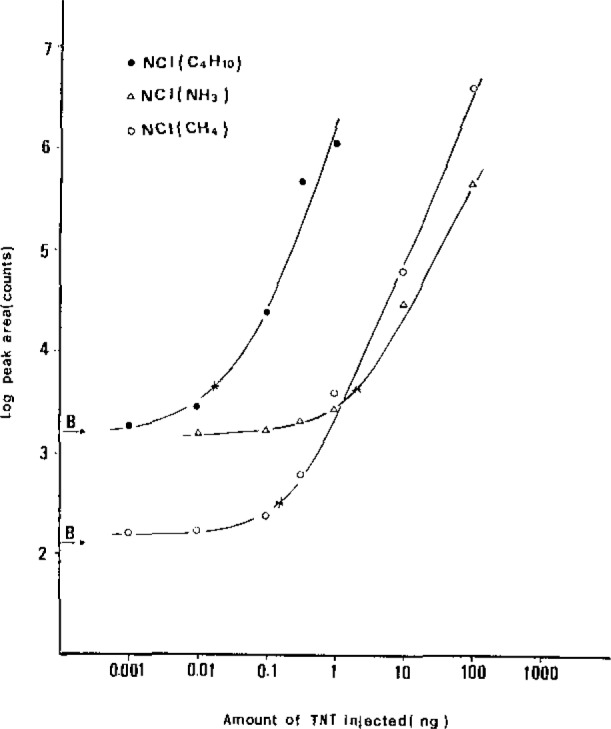
Calibration curves for the short capillary column NCI(i-C_4_H_10_)–SIM(227), NCI(CH_4_)–SIM(227) and NCI (NH_3_)–SIM(227) quantitation of TNT. B denotes the response of the acetone blank. * indicates the LOD.

The results of quantification of TNT in standards with a solids probe are shown in table 1. The LOD of both PCI and EI modes are at the same levels. In the NCI technique we always obtained a false signal at the same retention time as TNT. Therefore, the LOD of this technique cannot be defined. All the calibration curves were nonlinear as shown in figure 2. The lower response (i.e., sensitivity) at low amounts injected may be due to adsorption in the entire system, including the glassware and syringe.

## Conclusion

For the determination of TNT at trace levels, mass spectrometry has been shown to offer several advantages over other techniques. Comparing various MS monitoring modes with different reagent gases, the best monitoring mode for determination of TNT was shown to be isobutane negative chemical ionization with selected ion monitoring of the M^7^ of TNT at *m*/z 227. In a standard solution, the best TNT detection limit obtained with a short capillary column GC/NCI-SIM was 0.020 ng. Therefore, this NCI-SIM technique with its high sensitivity made it the preferred method for post-explosion residue analysis.

## Figures and Tables

**Table 1:** Comparison of different methods with short capillary column GC/MS and solids probe for determination of TNT (Source temperature: EI at 250 °C, CI at 150 °C)

Technique	Reagent gas		Ion monitored (*m*/*z*)	LOD (ng)	
	Type	Pressure (torr)		GS/MS	Solid-probe
EI			210	38	75
PCI	CH_4_	1.0	228	8.3	21
NCI	CH_4_	1.0	227	0.27	
PCI	i-C_4_H_10_	1.0	228	5.8	9.8
NCI	i-C_4_H_10_	1.0	227	0.02	
PCI	NH_3_	1.2	168	12	35
NCI	NH_3_	1.2	227	1.3	

## References

[1] Kraus S (1978).

[2] Yinon J (1982). Mass Spectrom Rev.

[3] Lee MR, Chang SC, Kao TS, Tang CP (1986). J Explos Propel, ROC.

[4] Yinon J (1980). J Forens Sci.

